# Supplementary material for: Ncl1-mediated metabolic rewiring critical during metabolic stress
Source: Life Sci Alliance. 2019 Aug 15;2(4):e201900360. doi: 10.26508/lsa.201900360 (PMC6696984; doi:10.26508/lsa.201900360)
Supplement: Supplementary file 8 [file LSA-2019-00360_TableS8.docx]

**Supplementary 8**: Optimized parameters for targeted metabolomics

| **Metabolite Name** | **Q1** | **Q3** | **DP (VOLTS)** | **EP (VOLTS)** | | **CE(VOLTS)** | **CXP(VOLTS)** |
| --- | --- | --- | --- | --- | --- | --- | --- |
| Alpha ketoglutarate | 145 | 101 | -60 | -8 | -13 | | -13 |
| Oxaloacetate | 131 | 113 | -40 | -10 | -23 | | -8 |
| Citrate/Isocitrate | 191 | 111 | -60 | -10 | -20 | | -10 |
| Succinate | 117 | 73 | -60 | -10 | -20 | | -10 |
| Ketoisocaproate (KIC) | 129 | 101 | -35 | -10 | -25 | | -11 |

Q1: Parental mass

Q2: Fragmented mass

DP- Declustering potential

EP-Entrance Potential

CE-Collision Energy

CXP: Collision Cell Exit Potential
